# Survey of Confidence and Knowledge in Managing Patellofemoral Pain among Physical Therapists in Saudi Arabia

**DOI:** 10.3390/healthcare12181891

**Published:** 2024-09-21

**Authors:** Marwan M. A. Aljohani, Abdulaziz Awali, Raghad Khalid Aljohani, Moiyad Saleh Aljehani, Yasir S. Alshehri

**Affiliations:** 1 Department of Physical Therapy, College of Medical Rehabilitation Sciences, Taibah University, Medina 42353, Saudi Arabia; yshehri@taibahu.edu.sa; 2 Department of Medical Rehabilitation Sciences, Umm Al-Qura University, Makkah 24382, Saudi Arabia; amawali@uqu.edu.sa (A.A.); msjehani@uqu.edu.sa (M.S.A.); 3 Department of Physical Therapy, King Salman Medical City, Ministry of Health, Medina 42319, Saudi Arabia; raghad.johani@gmail.com

**Keywords:** patellofemoral pain, clinical practice guidelines, evidence-based practice, physical therapist

## Abstract

Background: This study assessed the beliefs and knowledge of physical therapists in Saudi Arabia regarding the management of patellofemoral pain (PFP) and their alignment with current Clinical Practice Guidelines (CPGs). Methods: A cross-sectional survey was conducted, involving 111 licensed physical therapists actively treating PFP patients. The survey included questions about PFP risk factors, prognosis, diagnosis, and treatment, using a Likert scale to measure confidence and knowledge. Results: Readers have significantly greater confidence in the knowledge of managing patients with PFP following current CPGs (*p* < 0.01). No significant differences were observed between groups in the other items (*p* > 0.01). However, over 70% of respondents, irrespective of guideline familiarity, held beliefs about risk factors, prognosis, diagnosis, and treatment that were inconsistent with CPGs. Conclusions: These discrepancies highlight a significant knowledge gap that may affect patient care quality. Enhancing education and dissemination efforts regarding CPGs is essential to improve adherence to evidence-based practices among physical therapists in Saudi Arabia. To change practitioners’ preferences, attitudes, and beliefs, more targeted programs and interventions for knowledge dissemination and implementation should be provided.

## 1. Introduction

Patellofemoral pain (PFP) is common worldwide, impacting 23% to 29% of active and inactive adolescents and adults [1]. In Saudi Arabia, its prevalence is notably higher, ranging from 30.1% to 39% [2,3]. This substantial difference emphasizes the higher prevalence of PFP in the Saudi population compared to other regions worldwide. The primary symptoms involve pain around or directly behind the patella, which affects activities such as stair climbing, squatting, running, and sitting [4]. The long-term prognosis for PFP is concerning, as over 50% of those affected may continue to experience symptoms for as long as eight years after initial treatment [5]. Additionally, radiological evidence shows that 20% to 30% of adults could develop patellofemoral osteoarthritis [6]. Moreover, PFP may also lead to greater anxiety, depression, pain catastrophizing, fear of movement, lower health-related quality of life, and a reduced physical activity level in comparison to individuals without PFP [7,8]. Due to the high prevalence and significant impact of this condition, it is essential for clinicians and researchers to advance their knowledge of the most effective management techniques.

PFP is difficult to diagnose and treat, with about 74% of patients potentially having persistent pain 5–8 years after the diagnosis [5], with unwarranted variation in clinical practice being a significant issue. This practice variability in the management of PFP led to lower quality of care and poorer outcomes. An online survey of 99 physical therapists in the UK found that the majority of PTs indicated they would not prescribe an exercise if it caused pain to the patient [9]. However, previous research suggests that certain exercises can enhance treatment outcomes by improving self-efficacy and reducing pain sensitization [10,11]. Moreover, about 31% of physical therapists advised patients to discontinue leisure and/or sporting activities if experiencing any pain [9]. This approach could lead to unnecessary limitations on physical activity, potentially lead to a fear of movement and decreasing overall physical activity levels, which may negatively affect recovery and long-term musculoskeletal health.

The American Physical Therapy Association (APTA) has published a recent Clinical Practice Guideline (CPG) for PFP that focused on diagnosis, classification, examination, and a variety of intervention strategies to ensure evidence-based, effective treatment for patients [12,13]. Despite this, in the USA, the variability in managing PFP is still a barrier to effective care, which may lead to poorer patient outcomes [14]. Willy et al. found a significant inconsistency in the beliefs of physical therapists with the CPGs, particularly in risk factors and prognosis for PFP, regardless of whether they read the guidelines. However, readers of CPG have shown more accurate beliefs aligned with the current CPGs in treating PFP and a greater confidence in managing PFP compared to non-readers, therefore underscoring the importance of broadening the adoption of these guidelines to improve patient outcomes and reduce variability in treatment practices.

In Saudi Arabia, where the prevalence of PFP is high, it remains unclear how closely physical therapists adhere to the latest CPGs. Improving the clinical practices of physical therapists begins with evaluating their knowledge of guidelines, as this can substantially shape their attitudes and lead to enhanced clinical practices, resulting in better patient outcomes [15,16]. Therefore, a primary step in advancing the management of PFP involves evaluating physical therapists’ alignment with CPGs. Surveys are crucial for assessing the beliefs of physical therapists and identifying knowledge deficits in managing PFP. By comparing the insights of those who are familiar with the CPGs to those who are not, we can identify areas lacking in knowledge and develop more effective strategies for knowledge sharing in future CPGs for PFP. Therefore, the aim of this study was to assess and compare the beliefs of physical therapists in Saudi Arabia who are familiar with the CPGs versus those who are not, and to determine if their beliefs aligned with the current CPGs [13]. This research is crucial for optimizing treatment strategies for PFP in Saudi Arabia by ensuring that physical therapists’ beliefs and practices are in line with the latest CPGs, thereby promoting more consistent and effective outcomes for patients.

## 2. Materials and Methods

### 2.1. Study Design

This cross-sectional online survey was created according to the Checklist for reporting Results of the Internet E-Surveys Guideline recommendations [17]. It was conducted using Google Forms and distributed to licensed physical therapists in Saudi Arabia through social media. Data collection took place from 16 April to 5 May 2024. The online consent process provided participants with a description of the study, including its purpose, the researchers’ contact information, the estimated completion time, and a note that this survey was developed for licensed physical therapists currently working in Saudi Arabia and actively treating patients with PFP. Prior to participation, respondents provided informed consent by selecting “yes”. Participation was voluntary, anonymous, and confidential, with ethical approval granted by the Research Ethics Committee at Taibah University (approval number: CMR-PT-2024-07).

### 2.2. Survey Instrument

The survey used in the current study was adapted from a previous study after obtaining permission from the original authors of the questionnaire [18], which was developed based on a mixed-methods investigation conducted by Barton et al. [19]. The survey comprised Likert-based and open-ended questions to evaluate the confidence of physical therapists in Saudi Arabia and their knowledge of the diagnosis, prognosis, risk factors, and treatment strategies for patients with PFP (Supplementary Material). The survey was divided into four sections: (1) participant demographic, (2) confidence in managing PFP, (3) knowledge of risk factors, diagnosis, and prognosis, and (4) treatment strategies. All questions were developed based on the recommendations of the current CPG.

### 2.3. Participants Demographic

Demographic information gathered from participants included age, sex, highest level of education, occupational setting, and years of licensure or experience. Additional questions assessed whether participants treat patients with PFP, the number of PFP cases treated per year, whether participants have read the guidelines for managing PFP, and whether they have attended any workshops or lectures on the management of PFP.

### 2.4. Confidence in Managing PFP

Using 5-item Likert scales (strongly agree, agree, neither agree nor disagree, disagree, strongly disagree), participants rated their confidence in diagnosing PFP, recognizing its risk factors, managing the condition, providing appropriate treatment, and evaluating the availability and appropriateness of educational resources on general non-scientific websites (n = 6).

### 2.5. Knowledge of Risk Factors, Diagnosis, Prognosis, and Treatment Strategies

Participants rated their knowledge of risk factors (n = 11), diagnosis (n = 3), prognosis (n = 3), and treatment (n = 13) for PFP using 5-item Likert scales (strongly agree, agree, neither agree nor disagree, disagree, strongly disagree).

### 2.6. Statistical Analysis

Descriptive statistics were calculated, including the mean, standard deviation, and percentages, to provide an overview of the demographic and characteristics of the surveyed physical therapists. Mann–Whitney U tests were used to compare responses between readers and non-readers. The significance level was set at 0.01, which represents a conservative approach due to the large number of comparisons. All analyses were performed using SPSS software (version 28.0, IBM Corp., Armonk, NY, USA).

## 3. Results

### 3.1. Participants

**Figure 1:**
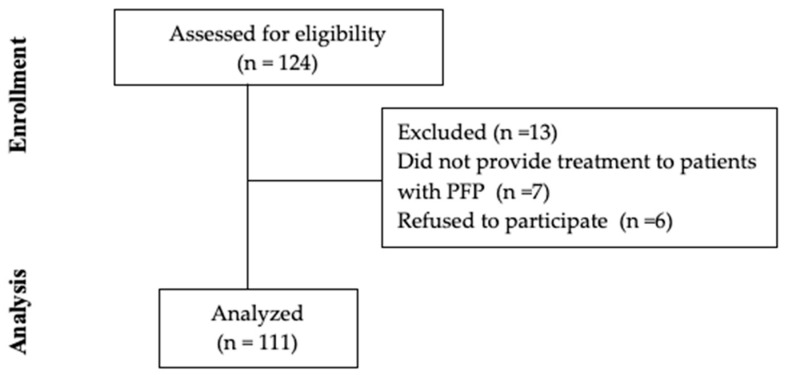
Diagram showing flow chart of participant enrollment, exclusion, and analysis.

The survey was accessed by a total of 124 individuals, out of which 118 voluntarily participated. Among these participants, seven individuals reported that they did not provide treatment to patients with PFP, and consequently, their data were excluded from the analysis (as illustrated in Figure 1). The demographics and characteristics of the remaining participants (n = 111) are summarized in Table 1. Based on their responses to the item “Have you read the latest Clinical Practice Guidelines for patellofemoral pain?”, participants were categorized into two groups: readers (n = 53) and non-readers (n = 58).

### 3.2. Confidence in Managing Patients with PFP

**Figure 2:**
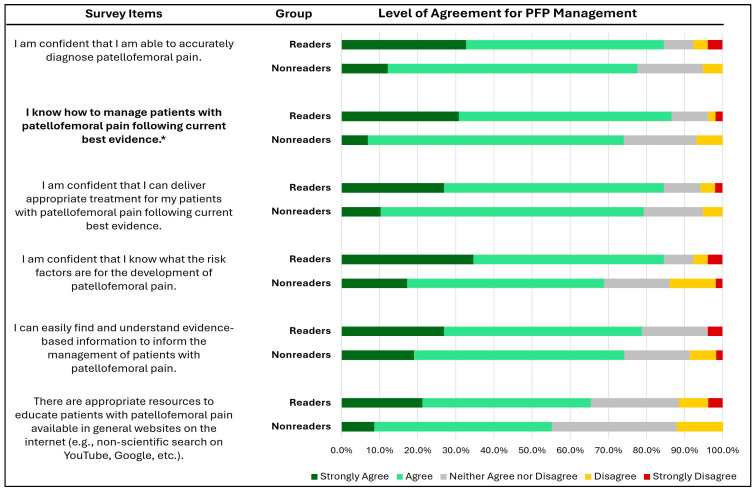
Level of agreement in the management of PFP between the surveyed physical therapists who have read (readers) vs. those who have not read (non-readers) the Clinical Practice Guidelines. * Significant at *p* < 0.01.

The confidence of the surveyed physical therapists in managing patients with PFP is illustrated in Figure 2. Overall, more than half of both readers and non-readers reported either strongly agreeing or agreeing on all items related to their confidence and knowledge in managing PFP. Across all items, the percentage of readers who strongly agree is consistently higher than that of non-readers. A significant difference was found between groups in the item related to knowledge in managing patients with PFP following the current best evidence (U = 1093.500, Z = −3.022, P = 0.003). No significant differences were observed between groups in the other five items (P = 0.018–0.31).

### 3.3. Knowledge of Risk Factors, Diagnosis, and Prognosis of PFP

**Figure 3:**
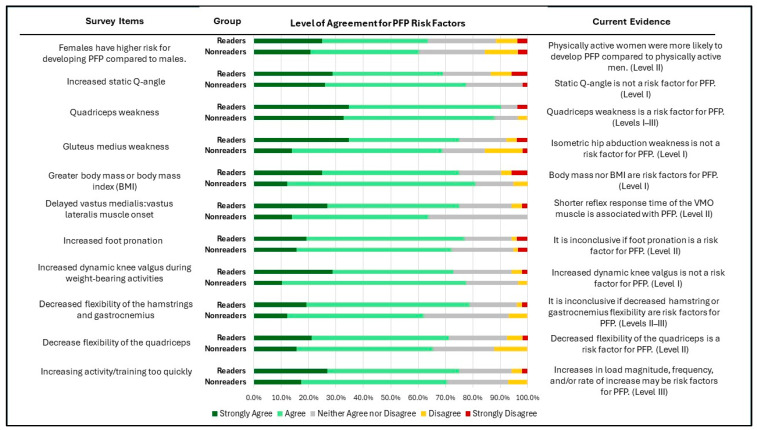
Level of agreement in PFP risk factors between the surveyed physical therapists who have read (readers) vs. those who have not read (non-readers) the Clinical Practice Guidelines.

**Figure 4:**
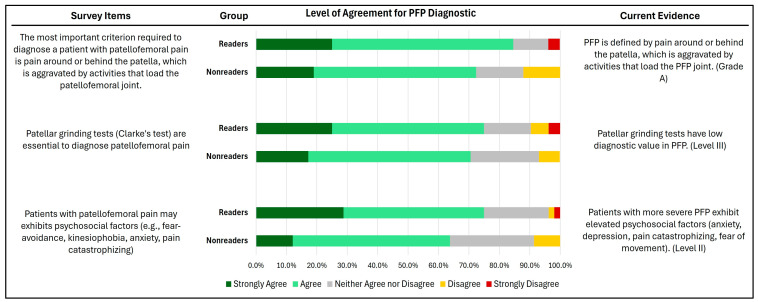
Level of agreement in PFP diagnostic between the surveyed physical therapists who have read (readers) vs. those who have not read (non-readers) the Clinical Practice Guidelines.

**Figure 5:**
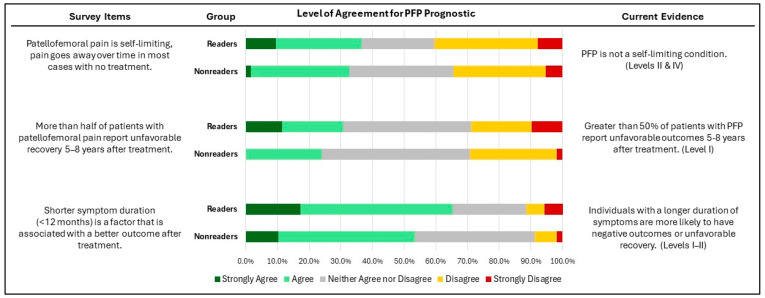
Level of agreement in PFP prognostic between the surveyed physical therapists who have read (readers) vs. those who have not read (non-readers) the Clinical Practice Guidelines.

Knowledge of risk factors, diagnosis, and prognosis of PFP among the surveyed physical therapists is shown in Figure 3, Figure 4 and Figure 5. Over 60% of both readers and non-readers agreed with statements related to risk factors and PFP diagnosis. Additionally, more than half of both groups acknowledged that shorter symptom duration (less than 12 months) is associated with better treatment outcomes. However, only a minority in both groups supported statements regarding the self-limiting nature of PFP and the belief that more than half of patients experience unfavorable recovery 5–8 years after treatment.

No significant differences were identified between groups across all items in the knowledge of risk factors, diagnosis, and prognosis of PFP (P = 0.03–0.94).

### 3.4. Knowledge of PFP Management

**Figure 6:**
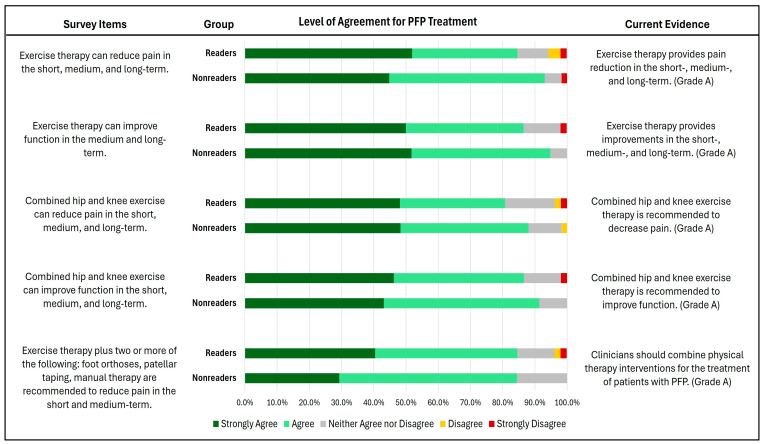
Level of agreement in exercise-related management of PFP between the surveyed physical therapists who have read (readers) vs. those who have not read (non-readers) the Clinical Practice Guidelines.

The results reflect a high level of agreement among both groups, with more than 80% reporting either strongly agreeing or agreeing on the positive effects of exercise therapy for managing PFP, both in terms of pain relief and functional improvement (Figure 6). However, no significant differences were observed between the groups (P = 0.48–0.94).

**Figure 7:**
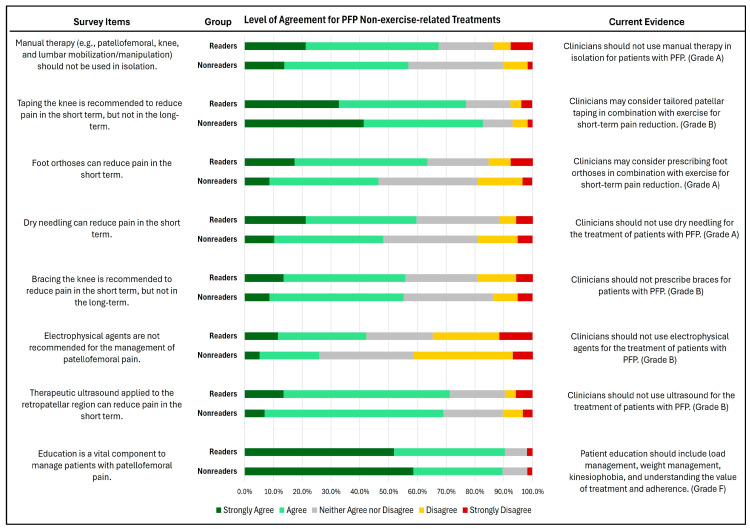
Level of agreement in non-exercise-related management of PFP between the surveyed physical therapists who have read (readers) vs. those who have not read (non-readers) the Clinical Practice Guidelines.

Both readers and non-readers recognized the importance of education in managing PFP, with 90.4% and 89.6% agreement, respectively (Figure 7). Additionally, over half of both groups agreed on the effectiveness of non-exercise-related treatments—such as therapeutic ultrasound, bracing, taping, and manual therapy—for PFP management.

However, only 25.9% of readers and 42.3% non-readers reported agreement regarding the recommendation of electrophysical agents for PFP management. No significant differences were noted between groups across all items (P = 0.08–0.95).

## 4. Discussion

The purpose of this cross-sectional survey was to assess the beliefs and knowledge of physical therapists working in Saudi Arabia and to determine whether their beliefs align with the current CPGs. Readers reported greater confidence and knowledge in following CPGs to manage patients with PFP compared to non-readers. Additionally, both groups showed a high level of agreement on the positive impact of exercise and education in treating PFP, regardless of whether they had read the guidelines. Despite this, the beliefs of the clinicians in both groups were misaligned with the current CPGs on several aspects regarding risk factors, prognosis, diagnosis, and passive treatments. These findings are important because they highlight the need for increased awareness and education among physical therapists in Saudi Arabia regarding the latest evidence-based recommendations for managing PFP. Improving clinicians’ knowledge and adherence to CPGs can lead to more effective and evidence-based care for patients with this common musculoskeletal condition. The majority of both readers and non-readers held either a bachelor’s or master’s degree and had comparable years of clinical practice. This suggests that they shared a similar foundational knowledge and clinical skill set. These findings might explain the absence of significant differences between the two groups, as their common academic backgrounds and clinical experience likely shaped their clinical perspectives and practices in similar ways.

The current study found that readers of CPGs have more confidence in their knowledge to follow the guidelines compared to non-readers. This is consistent with Willy et al. (2022), who also observed that readers of CPGs had greater confidence in implementing evidence-based practices for managing PFP, particularly in diagnosing, identifying risk factors, and treating PFP using CPGs [14]. However, the current study reveals that more than 70% of respondents, regardless of whether they had read the guidelines, strongly agree or agree that they have confidence in accurately diagnosing, identifying risk factors, delivering treatment based on best evidence, and understanding evidence-based information for managing PFP. Nevertheless, the confidence that non-readers have in managing PFP conditions does not necessarily mean that they manage PFP conditions based on evidence. Alshehri et al. (2017) found that the majority of physical therapists who participated in their study relied more on their clinical experiences compared to research articles [20]. A recent systematic review showed that many physical therapists do not follow clinical guidelines, and around 43% of them prescribe interventions that are not recommended by the clinical guidelines [21]. This suggests that the confidence in managing the conditions could be misleading and may not accurately reflect adherence to best practices or evidence-based care. Interestingly, more than 50% of both readers and non-readers believe that adequate resources are available to educate patients about PFP. However, there could be other potential barriers that limit the adherence to Clinical Practice Guidelines such as a lack of interest or support, or a lack of time [22]. Therefore, it is important to increase efforts to disseminate the CPGs through various channels, including workshops, webinars, and professional conferences, to ensure that all physical therapists are aware of the latest guidelines.

The current study shows that for knowledge of the risk factors, there was no significant difference between readers and non-readers. More than 60% of both groups identified being female, quadriceps muscle weakness, delayed vastus medialis/vastus lateralis muscle onset, decreased flexibility of the quadriceps, and increasing activity too quickly as risk factors, aligning with the CPGs. However, they also hold beliefs about other risk factors for PFP that do not align with the CPGs. Over 70% of respondents consider factors such as increased Q-angle, gluteus medius weakness, higher BMI, increased foot pronation, and increased dynamic knee valgus during weight-bearing activities as risk factors for PFP. Additionally, around 60% of physical therapists believe that decreased flexibility of the hamstrings and gastrocnemius are also risk factors. The CPGs do not recognize these as established risk factors, indicating a significant knowledge gap. This gap can lead to clinical practices focusing on non-evidence-based prevention strategies, such as using orthoses to reduce foot pronation or addressing dynamic knee angles. Clinicians may also emphasize strengthening the gluteus medius or improving the flexibility of the hamstrings and gastrocnemius to reduce the risk of developing PFP without supporting evidence. These findings are consistent with Willy et al. (2022), who also reported that physical therapists often misidentify risk factors for PFP and rely on non-evidence-based strategies [14]. They found similar discrepancies, noting that even among those who read the CPGs, there were significant misunderstandings regarding risk factors like increased Q-angle, gluteus medius weakness, increased BMI, and foot pronation. This highlights the need for increased education and the dissemination of evidence-based guidelines. By addressing these knowledge gaps, physical therapists can improve their prevention and management strategies for PFP.

Regarding the prognosis, the current study shows that more than 30% of clinicians in both groups believe PFP is self-limiting, and that pain will typically resolve over time without treatment. Additionally, about 30% of physical therapists strongly disagree or disagree that over 50% of patients with PFP report unfavorable recovery 5–8 years after treatment. These beliefs do not align with the CPGs and suggest that physical therapists might recommend ceasing exercises and delay the initiation of treatment, leading to negative outcomes and complicating future treatment. Also, reduced physical activity level in individuals with PFP can lead to greater anxiety, depression, pain catastrophizing, and fear of movement. Additionally, it contributes to a lower health-related quality of life [7,8]. Contrary to this belief, PFP is not self-limiting, and the timing of physical therapy is crucial [23]. Early intervention significantly reduces healthcare use, costs, and recurrence rates compared to delayed physical therapy [23,24]. Conversely, delaying physical therapy may worsen the prognosis [24,25]. Most clinicians in both groups believe that shorter symptom duration is associated with better outcomes after treatment, which aligns with the CPGs. Therefore, it is essential to educate physical therapists on the importance of early intervention to improve patient outcomes.

The present study shows no significant difference between readers and non-readers in the knowledge of diagnosing PFP. Most physical therapists believe that pain around or behind the patella, which worsens with activities that load the patellofemoral joint, is a key indicator of PFP. They also recognize that patients with PFP may experience psychosocial factors such as fear of avoidance, kinesiophobia, anxiety, and pain catastrophizing. These beliefs align with the CPGs, which state that PFP patients have retropatellar pain or pain around the patella that increases with squatting, stair climbing, running, or sitting. Additionally, individuals with PFP are less physically active than their healthy counterparts, taking fewer steps per day and engaging in less physical activity [7]. A low physical activity level is strongly associated with subjective function, pain, and fear-avoidance beliefs in individuals with PFP. Similarly, Will et al. found that the beliefs of physical therapists in the US are aligned with CPGs for the diagnosis of PFP [14]. However, contrary to the CPGs, clinicians in Saudi Arabia believe that Clarke’s test (i.e., patellar grinding test) is crucial for diagnosing PFP. This test has low sensitivity and moderate specificity and should not be used for diagnosing patients with PFP [26,27]. In contrast, the majority of PTs in the US strongly disagree or disagree that Clarke’s test is essential for diagnosing PFP [14]. This suggests physical therapists in Saudi Arabia may need further education to align their diagnostic practices with appropriate PFP diagnostic criteria, which is crucial for providing effective, evidence-based care. Addressing these knowledge gaps can lead to more accurate diagnoses and better outcomes for individuals with this common musculoskeletal condition.

Despite the differences between readers and non-readers in the confidence and knowledge of managing patients with PFP following current practices, both groups showed a high level of agreement regarding the effect of exercise therapy on pain and function. These responses align with current clinical guidelines that recommend exercise as the most effective intervention tool [13]. The absence of differences between the groups can be attributed to several reasons. First, moderate to strong evidence demonstrates the positive effects of exercise on decreasing pain and improving function in common musculoskeletal conditions [28,29]. Second, there is a shift in physical therapy practice from passive approaches towards active approaches such as exercise [30,31,32]. Therefore, this may explain why non-readers assume that exercise can decrease pain and improve function in people with PFP.

Similarly to exercise, both readers and non-readers agreed that education is a crucial element in the management of pain. This can be explained by the current transition from the biomedical approach in treating musculoskeletal conditions to the biopsychosocial approach [33], which incorporates patient education as a major element of the treatment for people with chronic pain [34]. Additionally, high levels of pain catastrophizing and fear of movement have been observed in people with PFP [8], and there is evidence that greater pain and kinesiophobia are associated with increased pain and disability [35] and with non-adherence to exercise [36]. Patient education has been shown to reduce pain and the psychological distress associated with it [34]. Thus, incorporating comprehensive patient education into treatment plans is essential for improving outcomes in individuals with PFP as with other chronic pain conditions.

Despite both readers and non-readers appreciating the effect of active treatments such as exercise for PFP, the majority of the sample agreed that passive treatments such as therapeutic ultrasound and dry needling can reduce pain in the short term. This agreement is not aligned with the recommendations of the CPGs, which advise against the use of therapeutic ultrasound and dry needling [13]. A Cochrane review showed that ultrasound has no clinically important effect on pain in individuals with PFP [37]. Similarly, the effect of dry needling on pain and disability is comparable to sham [38]. This could be explained by the fact that some therapists assume that passive intervention methods like dry needling can decrease pain, help retain patients, and recruit new ones [39]. There is also evidence that some patients prefer passive treatments like dry needling and electrical modalities and believe them to be effective [40]. Therefore, there remains a need for continued education and a dissemination of evidence-based guidelines, with an emphasis on avoiding interventions that lack supporting evidence.

Overall, this current study found that physical therapists have beliefs that are not aligned with the latest CPGs in several areas, including risk factors, prognosis, diagnosis, and the treatment of PFP. These inconsistencies between the knowledge of physical therapists and the CPGs may lead to ineffective prevention programs and suboptimal patient outcomes. Addressing these knowledge gaps through targeted educational programs, continuous professional development, and regular workshops is crucial to ensure that physical therapists provide evidence-based care for individuals with PFP. Improving the alignment between clinical practice and current guidelines can enhance the quality of care and lead to better long-term outcomes for patients with this musculoskeletal condition. Implementing strategies to bridge the gap between research and practice is essential for delivering the effective and comprehensive management of PFP. Although the data are localized to Saudi Arabia, the findings have broader implications, as similar gaps in knowledge and adherence to CPGs have been observed in other regions [14]. In addition, in many countries, it appears that many physical therapists do not consistently adhere to evidence-based guidelines in the management of musculoskeletal conditions [21]. Therefore, improving the alignment between clinical practice and current guidelines not only enhances the quality of care locally but also contributes to global efforts in standardizing the management of musculoskeletal conditions like PFP. Bridging the gap between research and practice is essential for delivering the effective and comprehensive management of PFP on an international scale.

One limitation of this study is the small sample size, which may restrict the generalizability of the findings. However, the participants were selected because they actively treat patients with PFP, which provides a more accurate representation of the beliefs and practices of physical therapists who manage patients with this condition. Another limitation is the use of an online survey, which may be subject to social desirability bias. Participants may have provided responses they believed were more socially acceptable, rather than accurately reflecting their true beliefs and practices. Despite this potential bias, surveys are important for assessing individual beliefs and identifying knowledge gaps. Future research could incorporate objective measures, such as clinical observations, to validate the findings and overcome the limitations of self-reported data. Another limitation of this study is the lack of specification regarding the version of the CPG referenced by participants. This may have led to variability in responses, as participants could have referred to different editions. Future research should clearly define the CPG version to ensure more consistent and reliable findings.

## 5. Conclusions

Readers have greater confidence in the management of PFP when following the CPGs compared to non-readers. However, a significant percentage of both groups hold beliefs that are not aligned with the CPGs regarding risk factors, prognosis, diagnosis, and the treatment of PFP. This may affect the quality of care given to patients. Therefore, it is essential to enhance education and dissemination efforts about the latest CPGs among physical therapists. Implementing comprehensive training programs, continuous professional development courses, and regular updates through workshops and webinars will help bridge the knowledge gap and ensure that all physical therapists provide evidence-based care. Additionally, to effectively change practitioners’ preferences, attitudes, and beliefs, it is crucial to implement targeted programs and interventions focused on knowledge dissemination and CPG implementation. This strategy will ultimately improve patient outcomes and standardize the quality of care across the profession.

## Figures and Tables

**Table 1:** Demographics and characteristics of the surveyed physical therapists *.

	Readers (n = 53)	Non-Readers (n = 58)	Total (n = 111)
Age, y	34.33 ± 7.28	34.29 ± 7.47	34.27 ± 7.33
Gender, n (%)			
Female	19 (35.8%)	23 (39.7%)	42 (37.8%)
Male	34 (64.2%)	35 (60.3%)	69 (62.2%)
Highest level of education, n (%)			
Bachelor’s	31 (58.5%)	29 (50.0%)	60 (54.1%)
Master’s	14 (26.4%)	19 (32.8%)	33 (29.7%)
DPT	1 (1.9%)	3 (5.2%)	4 (3.6%)
PhD	7 (13.2%)	7 (12.1%)	14 (12.6%)
Primary setting, n (%)			
Governmental hospital	30 (56.6%)	36 (62.1%)	66 (59.5%)
Private sectors	13 (24.5%)	12 (20.7%)	25 (22.5%)
Academia	6 (11.3%)	8 (13.8%)	14 (12.6%)
Professional sports team	3 (5.7%)	2 (3.5%)	5 (4.5%)
Others	1 (1.9%)	0 (0.0%)	1 (0.9%)
Years of qualifications as a physical therapist, n (%)			
<5 years	14 (26.4%)	14 (24.1%)	28 (25.2%)
5–10 years	17 (32.1%)	19 (32.8%)	36 (32.4%)
11–15 years	11 (20.8%)	10 (17.2%)	21 (18.9%)
16–20 years	6 (11.3%)	9 (15.5%)	15 (13.5%)
>20 years	5 (9.4%)	6 (10.3%)	11 (9.9%)
Number of patients with PFP treated per year, n (%)			
1–5	16 (30.2%)	23 (39.7%)	39 (35.1%)
6–15	16 (30.2%)	13 (22.4%)	29 (26.1%)
16–25	12 (22.6%)	4 (6.9%)	16 (14.4%)
≥26	4 (7.5%)	11 (19.0%)	15 (13.5%)
Unknown	5 (9.4%)	7 (12.1%)	12 (10.8%)

Abbreviations: DPT, Doctor of Physical Therapy; PhD, Doctor of Philosophy; PFP, patellofemoral pain. * Values are mean ± SD unless otherwise indicated.

## Data Availability

The data presented in this study are available on request from the corresponding author. The data are not publicly available due to respondents’ privacy.

## Supplementary Materials

The following supporting information can be downloaded at https://www.mdpi.com/article/10.3390/healthcare12181891/s1.

## References

[1] Smith B.E., Selfe J., Thacker D., Hendrick P., Bateman M., Moffatt F., Rathleff M.S., Smith T.O., Logan P. (2018). Incidence and Prevalence of Patellofemoral Pain: A Systematic Review and Meta-Analysis. PLoS ONE.

[2] Mohammad W.S., Elsais W.M. (2021). The Epidemiology of Patellofemoral Pain in Majmaah, Saudi Arabia. Asian J. Pharm. Res. Health Care.

[3] Aldharman S.S., Almuhammadi H.H., Madkhali A.Y., Alnami R.A., Alkadi M.A., Albalawi D.M., Alhamaid Y.A., Khired Z.A. (2022). Prevalence of Patellofemoral Pain and Knee Pain in the General Population of Saudi Arabia. Cureus.

[4] Lack S., Neal B., De Oliveira Silva D., Barton C. (2018). How to Manage Patellofemoral Pain-Understanding the Multifactorial Nature and Treatment Options. Phys. Ther. Sport..

[5] Lankhorst N.E., van Middelkoop M., Crossley K.M., Bierma-Zeinstra S.M.A., Oei E.H.G., Vicenzino B., Collins N.J. (2016). Factors That Predict a Poor Outcome 5-8 Years after the Diagnosis of Patellofemoral Pain: A Multicentre Observational Analysis. Br. J. Sports Med..

[6] Collins N.J., Oei E.H.G., de Kanter J.L., Vicenzino B., Crossley K.M. (2019). Prevalence of Radiographic and Magnetic Resonance Imaging Features of Patellofemoral Osteoarthritis in Young and Middle-Aged Adults With Persistent Patellofemoral Pain. Arthritis Care Res..

[7] Glaviano N.R., Baellow A., Saliba S. (2017). Physical Activity Levels in Individuals with and without Patellofemoral Pain. Phys. Ther. Sport..

[8] Maclachlan L.R., Collins N.J., Matthews M.L.G., Hodges P.W., Vicenzino B. (2017). The Psychological Features of Patellofemoral Pain: A Systematic Review. Br. J. Sports Med..

[9] Smith B.E., Hendrick P., Bateman M., Moffatt F., Rathleff M.S., Selfe J., Smith T.O., Logan P. (2017). Current Management Strategies for Patellofemoral Pain: An Online Survey of 99 Practising UK Physiotherapists. BMC Musculoskelet. Disord..

[10] Smith B.E., Hendrick P., Smith T.O., Bateman M., Moffatt F., Rathleff M.S., Selfe J., Logan P. (2017). Should Exercises Be Painful in the Management of Chronic Musculoskeletal Pain? A Systematic Review and Meta-Analysis. Br. J. Sports Med..

[11] Smith B.E., Hendrick P., Bateman M., Holden S., Littlewood C., Smith T.O., Logan P. (2019). Musculoskeletal Pain and Exercise-Challenging Existing Paradigms and Introducing New. Br. J. Sports Med..

[12] Wallis J.A., Roddy L., Bottrell J., Parslow S., Taylor N.F. (2021). A Systematic Review of Clinical Practice Guidelines for Physical Therapist Management of Patellofemoral Pain. Phys. Ther..

[13] Willy R.W., Hoglund L.T., Barton C.J., Bolgla L.A., Scalzitti D.A., Logerstedt D.S., Lynch A.D., Snyder-Mackler L., McDonough C.M. (2019). Patellofemoral Pain. J. Orthop. Sports Phys. Ther..

[14] Willy R.W., Hoglund L.T., Glaviano N.R., Bolgla L.A., Bazett-Jones D.M. (2022). Survey of Confidence and Knowledge to Manage Patellofemoral Pain in Readers versus Nonreaders of the Physical Therapy Clinical Practice Guideline. Phys. Ther. Sport..

[15] Woolf S.H. (1993). Practice Guidelines: A New Reality in Medicine. III. Impact on Patient Care. Arch. Intern. Med..

[16] Cabana M.D., Rand C.S., Powe N.R., Wu A.W., Wilson M.H., Abboud P.A., Rubin H.R. (1999). Why Don’t Physicians Follow Clinical Practice Guidelines? A Framework for Improvement. JAMA.

[17] Eysenbach G. (2004). Improving the Quality of Web Surveys: The Checklist for Reporting Results of Internet E-Surveys (CHERRIES). J. Med. Internet Res..

[18] Zambarano E.K., Bazett-Jones D.M., de Oliveira Silva D., Barton C.J., Glaviano N.R. (2022). Confidence and Knowledge of Athletic Trainers in Managing Patellofemoral Pain. J. Athl. Train..

[19] Barton C.J., Ezzat A.M., Bell E.C., Rathleff M.S., Kemp J.L., Crossley K.M. (2022). Knowledge, Confidence and Learning Needs of Physiotherapists Treating Persistent Knee Pain in Australia and Canada: A Mixed-Methods Study. Physiother. Theory Pract..

[20] Alshehri M.A., Alalawi A., Alhasan H., Stokes E. (2017). Physiotherapists’ Behaviour, Attitudes, Awareness, Knowledge and Barriers in Relation to Evidence-Based Practice Implementation in Saudi Arabia. Int. J. Evid. Based Healthc..

[21] Zadro J., O’Keeffe M., Maher C. (2019). Do Physical Therapists Follow Evidence-Based Guidelines When Managing Musculoskeletal Conditions? Systematic Review. BMJ Open.

[22] Paci M., Faedda G., Ugolini A., Pellicciari L. (2021). Barriers to Evidence-Based Practice Implementation in Physiotherapy: A Systematic Review and Meta-Analysis. Int. J. Qual. Health Care.

[23] van Middelkoop M., van der Heijden R.A., Bierma-Zeinstra S.M.A. (2017). Characteristics and Outcome of Patellofemoral Pain in Adolescents: Do They Differ From Adults?. J. Orthop. Sports Phys. Ther..

[24] Young J.L., Snodgrass S.J., Cleland J.A., Rhon D.I. (2021). Timing of Physical Therapy for Individuals with Patellofemoral Pain and the Influence on Healthcare Use, Costs and Recurrence Rates: An Observational Study. BMC Health Serv. Res..

[25] Matthews M., Rathleff M.S., Claus A., McPoil T., Nee R., Crossley K., Vicenzino B. (2017). Can We Predict the Outcome for People with Patellofemoral Pain? A Systematic Review on Prognostic Factors and Treatment Effect Modifiers. Br. J. Sports Med..

[26] Nunes G.S., Stapait E.L., Kirsten M.H., de Noronha M., Santos G.M. (2013). Clinical Test for Diagnosis of Patellofemoral Pain Syndrome: Systematic Review with Meta-Analysis. Phys. Ther. Sport..

[27] Nijs J., Van Geel C., Van der auwera C., Van de Velde B. (2006). Diagnostic Value of Five Clinical Tests in Patellofemoral Pain Syndrome. Man. Ther..

[28] De la Corte-Rodriguez H., Roman-Belmonte J.M., Resino-Luis C., Madrid-Gonzalez J., Rodriguez-Merchan E.C. (2024). The Role of Physical Exercise in Chronic Musculoskeletal Pain: Best Medicine-A Narrative Review. Healthcare.

[29] Babatunde O.O., Jordan J.L., Van der Windt D.A., Hill J.C., Foster N.E., Protheroe J. (2017). Effective Treatment Options for Musculoskeletal Pain in Primary Care: A Systematic Overview of Current Evidence. PLoS ONE.

[30] White N.T., Delitto A., Manal T.J., Miller S. (2015). The American Physical Therapy Association’s Top Five Choosing Wisely Recommendations. Phys. Ther..

[31] Luna E.G., Hanney W.J., Rothschild C.E., Kolber M.J., Liu X., Masaracchio M. (2019). The Influence of an Active Treatment Approach in Patients With Low Back Pain: A Systematic Review. Am. J. Lifestyle Med..

[32] Fritz J.M., Cleland J.A., Brennan G.P. (2007). Does Adherence to the Guideline Recommendation for Active Treatments Improve the Quality of Care for Patients with Acute Low Back Pain Delivered by Physical Therapists?. Med. Care.

[33] Lin I., Wiles L., Waller R., Goucke R., Nagree Y., Gibberd M., Straker L., Maher C.G., O’Sullivan P.P.B. (2020). What Does Best Practice Care for Musculoskeletal Pain Look like? Eleven Consistent Recommendations from High-Quality Clinical Practice Guidelines: Systematic Review. Br. J. Sports Med..

[34] Louw A., Zimney K., Puentedura E.J., Diener I. (2016). The Efficacy of Pain Neuroscience Education on Musculoskeletal Pain: A Systematic Review of the Literature. Physiother. Theory Pract..

[35] Domenech J., Sanchis-Alfonso V., López L., Espejo B. (2013). Influence of Kinesiophobia and Catastrophizing on Pain and Disability in Anterior Knee Pain Patients. Knee Surg. Sports Traumatol. Arthrosc..

[36] Zhou Y., Gao W., Gao S., Guo X., Liu M., Cao C. (2023). Pain Catastrophizing, Kinesiophobia and Exercise Adherence in Patients After Total Knee Arthroplasty: The Mediating Role of Exercise Self-Efficacy. J. Pain. Res..

[37] Brosseau L., Casimiro L., Welch V., Milne S., Shea B., Judd M., Wells G.A., Tugwell P., Brosseau L. (2001). Therapeutic Ultrasound for Treating Patellofemoral Pain Syndrome. Cochrane Database of Systematic Reviews.

[38] Sutlive T.G., Golden A., King K., Morris W.B., Morrison J.E., Moore J.H., Koppenhaver S. (2018). Short-term effects of trigger point dry needling on pain and disability in subjects with patellofemoral pain syndrome. Int. J. Sports Phys. Ther..

[39] Ijaz N., Welsh S., Boon H. (2021). A Mixed-Methods Survey of Physiotherapists Who Practice Acupuncture and Dry Needling in Ontario, Canada: Practice Characteristics, Motivations, and Professional Outcomes. BMC Complement. Med. Ther..

[40] Bernhardsson S., Larsson M.E.H., Johansson K., Öberg B. (2017). “In the Physio We Trust”: A Qualitative Study on Patients’ Preferences for Physiotherapy. Physiother. Theory Pract..

