# Supplementary material for: Survey of Confidence and Knowledge in Managing Patellofemoral Pain among Physical Therapists in Saudi Arabia
Source: Healthcare (Basel). 2024 Sep 21;12(18):1891. doi: 10.3390/healthcare12181891 (PMC11431332; doi:10.3390/healthcare12181891)
Supplement: Supplementary file 1 [file healthcare-12-01891-s001.zip › healthcare-3134917-supplementary.pdf]

Survey of Confidence and Knowledge in Managing Patellofemoral Pain among Physical Therapists in Saudi Arabia

Section 1: Demographics

1. Do you work in Saudi Arabia?

- Yes
- No

If the participant chooses "No", then end the survey.

---

2. Age

- [Enter age between 0-100]

---

3. Sex

- Male
- Female

---

4. Highest level of education *(required)*

- Bachelor's
- Master's
- Doctor of Physical Therapy
- PhD

---

5. Primary Setting *(required)*

- Professional team sports
- Governmental hospital
- Academia
- Private sectors
- Other (please specify)

6. How long have you been qualified as a physical therapist? *(required)*

- Less than 5 years
- 5-10 years
- 11-15 years
- 16-20 years
- More than 20 years

7. Have you read the latest Clinical Practice Guidelines for Patellofemoral pain? *(required)*

- Yes
- No

8. Have you attended any recent workshop or lecture that discussed the management of Patellofemoral pain ? *(required)*

- Yes
- No

9. Are you currently treating a patient with Patellofemoral pain? *(required)*

- Yes
- No

10. Number of patients with Patellofemoral pain treated per year *(required)*

- 0-5
- 6-15
- 16-25
- 26 or more
- Unknown

Section 2: Level of Agreement for Management of PFP

Please indicate your level of agreement with the following statements:

11. I am confident that I am able to accurately diagnose Patellofemoral pain. *(required)*

- Strongly disagree
- Disagree
- Neither agree nor disagree
- Agree
- Strongly agree

---

12. I know how to manage patients with PFP following current best evidence. *(required)*

- Strongly disagree
- Disagree
- Neither agree nor disagree
- Agree
- Strongly agree

---

13. I am confident that I can deliver appropriate treatment for my patients with Patellofemoral pain following current best evidence. *(required)*

- Strongly disagree
- Disagree
- Neither agree nor disagree
- Agree
- Strongly agree

---

14. I am confident that I know what the risk factors are for the development of Patellofemoral pain. *(required)*

- Strongly disagree
- Disagree
- Neither agree nor disagree
- Agree
- Strongly agree

---

15. I can easily find and understand evidence-based information to inform the management of patients with PFP.

- Strongly disagree
- Disagree
- Neither agree nor disagree
- Agree
- Strongly agree

---

16. There are appropriate resources to educate patients with PFP available in general websites on the internet (e.g., non-scientific search on YouTube, Google, etc.).

- Strongly disagree
- Disagree
- Neither agree nor disagree
- Agree
- Strongly agree

Section 3: Risk Factors, Diagnosis and prognosis of patellofemoral pain

17. Females have higher risk for developing patellofemoral pain compared to males. *(required)*

- Strongly disagree
- Disagree
- Neither agree nor disagree
- Agree
- Strongly agree

---

18. Increased static Q-angle is considered as a risk factor for patellofemoral pain. *(required)*

- Strongly disagree
- Disagree
- Neither agree nor disagree
- Agree
- Strongly agree

19. Quadriceps weakness is considered as a risk factor for patellofemoral pain. *(required)*

- Strongly disagree
- Disagree
- Neither agree nor disagree
- Agree
- Strongly agree

---

20. Gluteus medius weakness is considered as a risk factor for patellofemoral pain. *(required)*

- Strongly disagree
- Disagree
- Neither agree nor disagree
- Agree
- Strongly agree

---

21. Greater body mass or body mass index (BMI) is considered as a risk factor for patellofemoral pain. *(required)*

- Strongly disagree
- Disagree
- Neither agree nor disagree
- Agree
- Strongly agree

---

22. Delayed vastus medialis:vastus lateralis muscle onset is considered a risk factor for patellofemoral pain. *(required)*

- Strongly disagree
- Disagree
- Neither agree nor disagree
- Agree
- Strongly agree

---

23. Increased foot pronation is considered as a risk factor for patellofemoral pain.

- Strongly disagree
- Disagree
- Neither agree nor disagree
- Agree
- Strongly agree

---

24. Increased dynamic knee valgus during weight-bearing activities is considered as a risk factor for patellofemoral pain.*(required)*

- Strongly disagree
- Disagree
- Neither agree nor disagree
- Agree
- Strongly agree

---

25. Decreased flexibility of the hamstrings and gastrocnemius is considered as a risk factor for patellofemoral pain. *(required)*

- Strongly disagree
- Disagree
- Neither agree nor disagree
- Agree
- Strongly agree

---

26. Decreased flexibility of the quadriceps is considered as a risk factor for PFP. *(required)*

- Strongly disagree
- Disagree
- Neither agree nor disagree
- Agree
- Strongly agree

---

27. Increasing activity/training too quickly is considered as a risk factor for PFP. *(required)*

- Strongly disagree
- Disagree

- Neither agree nor disagree
- Agree
- Strongly agree

---

28. The most important criterion required to diagnose a patient with patellofemoral pain is pain around or behind the patella, which is aggravated by activities that load the patellofemoral joint. *(required)*

- Strongly disagree
- Disagree
- Neither agree nor disagree
- Agree
- Strongly agree

---

29. Patellar grinding tests (Clarke's test) are essential to diagnose patellofemoral pain. *(required)*

- Strongly disagree
- Disagree
- Neither agree nor disagree
- Agree
- Strongly agree

---

30. Patients with patellofemoral pain may exhibit psychosocial factors (e.g., fear-avoidance, kinesiophobia, anxiety, pain catastrophizing). *(required)*

- Strongly disagree
- Disagree
- Neither agree nor disagree
- Agree
- Strongly agree

31. PFP is self-limiting; pain goes away over time in most cases with no treatment. *(required)*

- Strongly disagree
- Disagree
- Neither agree nor disagree

- Agree
- Strongly agree

---

32. More than half of patients with patellofemoral pain report unfavorable recovery 5-8 years after treatment. *(required)*

- Strongly disagree
- Disagree
- Neither agree nor disagree
- Agree
- Strongly agree

---

33. Shorter symptom duration (<12 months) is a factor that is associated with a better outcome after treatment.

- Strongly disagree
- Disagree
- Neither agree nor disagree
- Agree
- Strongly agree

Section 5: Treatment of patellofemoral pain

34. Exercise therapy can reduce pain in the short, medium, and long-term. *(required)*

- Strongly disagree
- Disagree
- Neither agree nor disagree
- Agree
- Strongly agree

---

35. Exercise therapy can improve function in the medium and long-term. *(required)*

- Strongly disagree
- Disagree
- Neither agree nor disagree
- Agree
- Strongly agree

36. Combined hip and knee exercise can reduce pain in the short, medium, and long-term. *(required)*

- Strongly disagree
- Disagree
- Neither agree nor disagree
- Agree
- Strongly agree

37. Combined hip and knee exercises can improve function in the short, medium, and long-term. *(required)*

- Strongly disagree
- Disagree
- Neither agree nor disagree
- Agree
- Strongly agree

38. Exercise therapy plus two or more of the following: foot orthoses, patellar taping, manual therapy are recommended to reduce pain in the short and medium-term. *(required)*

- Strongly disagree
- Disagree
- Neither agree nor disagree
- Agree
- Strongly agree

39. Manual therapy (e.g., patellofemoral, knee, and lumbar mobilization/manipulation) should not be used in isolation. *(required)*

- Strongly disagree
- Disagree
- Neither agree nor disagree
- Agree
- Strongly agree

40. Taping the knee is recommended to reduce pain in the short term, but not in the long-term. *(required)*

- Strongly disagree
- Disagree
- Neither agree nor disagree
- Agree
- Strongly agree

---

41. Foot orthoses can reduce pain in the short term. *(required)*

- Strongly disagree
- Disagree
- Neither agree nor disagree
- Agree
- Strongly agree

---

42. Dry needling can reduce pain in the short term. *(required)*

- Strongly disagree
- Disagree
- Neither agree nor disagree
- Agree
- Strongly agree

---

43. Bracing the knee is recommended to reduce pain in the short term, but not in the long-term.

- Strongly disagree
- Disagree
- Neither agree nor disagree
- Agree
- Strongly agree

---

44. Electrophysical agents are not recommended for the management of patellofemoral pain. *(required)*

- Strongly disagree

- Disagree
- Neither agree nor disagree
- Agree
- Strongly agree

---

45. Therapeutic ultrasound applied to the retropatellar region can reduce pain in the short term. *(required)*

- Strongly disagree
- Disagree
- Neither agree nor disagree
- Agree
- Strongly agree

---

46. Education is a vital component to manage patients with patellofemoral pain. *(required)*

- Strongly disagree
- Disagree
- Neither agree nor disagree
- Agree
- Strongly agree

End of Survey
